# Assessment of the COVID-19 pandemic progression in Ecuador through seroprevalence analysis of anti-SARS-CoV-2 IgG/IgM antibodies in blood donors

**DOI:** 10.3389/fcimb.2024.1373450

**Published:** 2024-06-21

**Authors:** Aníbal Gaviria, Rafael Tamayo-Trujillo, Elius Paz-Cruz, Santiago Cadena-Ullauri, Patricia Guevara-Ramírez, Viviana A. Ruiz-Pozo, Francisco Cevallos, Víctor Aguirre-Tello, Karla Risueño, Martha Paulina Yánez, Alejandro Cabrera-Andrade, Ana Karina Zambrano

**Affiliations:** ^1^ Laboratorio de Genética, Centros Médicos Especializados Cruz Roja Ecuatoriana, Quito, Ecuador; ^2^ Hemocentro Nacional, Cruz Roja Ecuatoriana, Quito, Ecuador; ^3^ Centro de Investigación Genética y Genómica, Facultad de Ciencias de la Salud Eugenio Espejo, Universidad UTE, Quito, Ecuador; ^4^ Grupo de Bio-Quimioinformática, Universidad de Las Américas, Quito, Ecuador; ^5^ Escuela de Enfermería, Facultad de Ciencias de la Salud, Universidad de Las Américas, Quito, Ecuador

**Keywords:** COVID-19, seroprevalence, Ecuador, IgG, IgM

## Abstract

**Introduction:**

Coronavirus Disease 2019 (COVID-19) is a severe respiratory illness caused by the RNA virus SARS-CoV-2. Globally, there have been over 759.4 million cases and 6.74 million deaths, while Ecuador has reported more than 1.06 million cases and 35.9 thousand deaths. To describe the COVID-19 pandemic impact and the vaccinations effectiveness in a low-income country like Ecuador, we aim to assess the seroprevalence of IgG and IgM antibodies against SARS-CoV-2 in a sample from healthy blood donors at the Cruz Roja Ecuatoriana.

**Methods:**

The present seroprevalence study used a lateral flow immunoassay (LFIA) to detect anti-SARS-CoV-2 IgG and IgM antibodies in months with the highest confirmed case rates (May 2020; January, April 2021; January, February, June, July 2022) and months with the highest vaccination rates (May, June, July, August, December 2021) in Quito, Ecuador. The IgG and IgM seroprevalence were also assessed based on sex, age range, blood type and RhD antigen type. The sample size was 8,159, and sampling was performed based on the availability of each blood type.

**Results:**

The results showed an overall IgG and IgM seroprevalence of 47.76% and 3.44%, respectively. There were no differences in IgG and IgM seroprevalences between blood groups and sex, whereas statistical differences were found based on months, age range groups, and RhD antigen type. For instance, the highest IgG seroprevalence was observed in February 2022 and within the 17-26 years age range group, while the highest IgM seroprevalence was in April 2021 and within the 47-56 years age range group. Lastly, only IgG seroprevalence was higher in RhD+ individuals while IgM seroprevalence was similar across RhD types.

**Discussion:**

This project contributes to limited data on IgG and IgM antibodies against SARS-CoV-2 in Ecuador. It suggests that herd immunity may have been achieved in the last evaluated months, and highlights a potential link between the RhD antigen type and COVID-19 susceptibility. These findings have implications for public health strategies and vaccine distribution not only in Ecuador but also in regions with similar characteristics.

## Introduction

Coronavirus disease 2019 (COVID-19) is a severe respiratory illness caused by the RNA virus known as SARS-CoV-2 (severe acute respiratory syndrome coronavirus 2) (Jamison et al., 2022). The primary COVID-19 symptoms are fever, persistent cough, shortness of breath, insomnia, and anosmia. The severity of these symptoms could vary depending on age and the presence of comorbidities (Lamers and Haagmans, 2022).

Infected individuals may either be asymptomatic or exhibit mild to moderate respiratory symptoms. However, in some cases, patients could develop systemic hyperinflammation, which may lead to fatal acute respiratory distress syndrome (ARDS). This hyperinflammation involves the release of several proinflammatory proteins, and high concentrations of inflammatory markers (Lamers and Haagmans, 2022).

After the notification of a COVID-19 spread outside China (January 2020), a global surveillance program was launched to monitor COVID-19 cases, related deaths, risk factors, and vaccination status (Allan et al., 2022). Worldwide, until April 2024, more than 775.3 million COVID-19 cases (World Health Organization, 2024a) and more than 6.74 million deaths (World Health Organization, 2024b) have been recorded. The vaccination coverage shows that more than 13.5 billion doses have been administrated around the world until May 2024, although only 32.8% of people in low-income countries have received at least one dose (Mathieu et al., 2024). Lastly, a systematic review found that the global anti-SARS-CoV-2 seroprevalence, up until September 2021, reached at 59.2% (Bergeri et al., 2022).

In the case of Ecuador, the Public Health Ministry (MSP, by its Spanish acronym) announced the identification of the first COVID-19 patient on February 29, 2020 (Vallejo-Janeta et al., 2022). Subsequently, on March 16, 2020, they announced the beginning of restrictive measures to mitigate the spread of the disease within the country (Ministerio de Salud Pública, 2020). Furthermore, the Our World in Data database has reported more than 1.08 million COVID-19 cases and more than 36.04 thousand deaths until Apryl 2024 (Mathieu et al., 2020a; Mathieu et al., 2020b). It is noteworthy that, to date, no country-wide seroprevalence studies have been performed in Ecuador. The COVID-19 impact on the Ecuadorian population could be underestimated, due to limited number of available tests, insufficient personnel, and scarce funding, especially at the onset of the pandemic (Ortiz-Prado et al., 2021; Orellana-Manzano et al., 2023).

Moreover, research have investigated the association between blood group and susceptibility to SARS-CoV-2 infection (Fan et al., 2020; Samra et al., 2021; Shibeeb and Khan, 2022). Some studies have suggested that the blood group O could have a protective effect due to the natural presence of anti-A and anti-B antibodies. Conversely, blood types A (presence of anti-B antibodies), B (presence of anti-A antibodies), or AB (no antibodies presence) may be potential COVID-19 risk factors, as these antigens might facilitate SARS-CoV-2 entry into the cell (Barnkob et al., 2020; Tamayo-Velasco et al., 2022). However, it is important to note that the available evidence on this topic has been highly heterogeneous (Zietz et al., 2020; Almadhi et al., 2021; Kabrah et al., 2021; Samra et al., 2021).

COVID-19 diagnosis is mainly performed using nucleic acid testing through RT-qPCR or via viral antigen detection. Moreover, another approach for oCOVID-19 diagnosis is antibody detection utilizing serologic assays; however, it may be difficult, due to it requires detectable antibody titter levels, which takes time after viral exposure (Ortiz-Prado et al., 2020). However, serological tests are useful to detect individuals who have been either infected with the virus at some point or vaccinated against SARS-CoV2 (Moya Rios do Vale et al., 2022). Due to their simplicity, cost-effectiveness, and rapid results, serological tests are a viable option for evaluating the status of anti-SARS-CoV-2 antibodies (Ortiz-Prado et al., 2020).

Several countries have conducted serological studies for SARS-CoV-2 surveillance using donor blood samples (Borges et al., 2020; Filho et al., 2020; Garcia-Basteiro et al., 2020; Rodríguez-Vidales et al., 2021; Siller et al., 2021; Kaboré et al., 2023). The analysis of the IgM seroprevalence could provide information about the SARS-CoV-2 infection status on healthy blood donors after donation triage (Borges et al., 2020; Garcia-Basteiro et al., 2020; Chaves et al., 2022). Moreover, the IgM seroprevalence indicates the proportion of individuals with active or recent SARS-CoV-2 infections (Ortiz-Prado et al., 2020; Alibolandi et al., 2022; Fraussen, 2022). Furthermore, a metanalysis by Fox et al. (2022) mentions that IgM antibodies could also appear late during acute illness. This suggests that it may not only be a definitive indicator of current infections but also past infections (Fox et al., 2022).

On the other hand, IgG seroprevalence could show the immune state at community level due to infection exposure or vaccination programs (Jaiswal et al., 2022). Furthermore, the presence of high IgG antibody rates can indicate if herd immunity has been reached (Ali et al., 2023).

In this research article, a seroprevalence study was performed using a lateral flow immunoassay (LFIA), a type of serological test that identifies anti-SARS-CoV-2 IgG and IgM antibodies. This study was conducted in seemingly healthy individuals who donated blood at the Cruz Roja Ecuatoriana between 2020 and 2022. The association between blood groups, different time points, age, sex, and SARS-CoV-2 seroprevalence was evaluated.

## Materials and methods

### Ethical considerations

All experiments were performed in compliance with relevant laws, institutional guidelines, and the ethical standards of the Declaration of Helsinki. The study was initiated after sample collection at the Hemocentro of the Cruz Roja Ecuatoriana and each sample’s data was anonymized to ensure that no identifiable information was included. This study was approved by the Human Beings Research Ethics Committee of Universidad UTE (code number: CEISH-2022-033). Informed consent was not necessary given that the research involved material or data contained in biobanks or similar repositories, as outlined in item 32 of the Informed Consent section of the Helsinki Declaration (World Medical Association, 2022).

### Study design

A SARS-CoV-2 IgG/IgM seroprevalence study was conducted using samples from 2020 to 2022 as a collaborative project between three Ecuadorian institutions: the Hemocentro Nacional of the Cruz Roja Ecuatoriana, Centros Médicos Especializados Cruz Roja Ecuatoriana, and the Centro de Investigación Genética y Genómica of the Universidad UTE. The samples analyzed came from blood donors whose well-being was assessed through a survey before donation at the Hemocentro Nacional of the Cruz Roja Ecuatoriana. The survey is designed to evaluate the donor’s health and eligibility for blood donation.

The sample size was 8,159, and it was determined based on the availability of each blood type and different periods according to the highest confirmed cases and vaccination rates (**Supplementary Table 1**). Samples were collected during: *i)* months with the highest confirmed case rates [May 2020 (*n*:797), January 2021 (*n*:891), April 2021 (*n*:980), January 2022 (*n*:412), February 2022 (n:413), June 2022 (n:409), July 2022 (*n*:409)], and *ii)* months with the highest vaccination rates, First dose: [May 2021 (n:933), June 2021 (*n*:933), July 2021 (*n*:781); Second dose: August 2021 (*n*:681); Booster dose: December 2021 (*n*:520)] (Ministerio de Salud Pública, 2020; Ministerio de Salud Pública del Ecuador, 2020; Ministerio de Salud Pública, 2022). Apart from the 8.159 samples, 1.018 samples were collected from the period between January and March 2020, just to corroborate the absence of IgG/IgM antibodies before the beginning of the restrictive measures in Ecuador.

The study included samples from all ABO blood groups (A, B, AB, and O) based on their prevalence. In Ecuador, the prevalence rates were as follows: 75.46% for blood type O, 17.14% for type A, 6.69% for type B, and 0.68% for type AB (Núñez Cifuentes, 2022). Additionally, the samples were categorized based on the presence of the Rhesus D antigen. Notably, Ecuador has one of the highest percentages of the O RhD+ blood type (Alkout and Alkout, 2020); therefore, this blood group (O [n:3971]) was the most sampled, followed by A [n:2280], B [n:1524], and AB [n:384]. Demographic information, such as age and sex, was collected. The ages were grouped into five categories.

### Anti-SARS-CoV-2 antibody detection

The assessment of anti-SARS-CoV-2 IgG/IgM antibodies was performed using the LFIA assay Rapid 2019-nCoV IgG/IgM Combo Test Card (Colloidal Gold) manufactured by BioDetect Biotechnology, Xiamen, China. The manufacturer indicates values of 95% and 92.3% for IgG and IgM specificities and 100% and 90.4% for IgG and IgM sensibility. Moreover, the test showed no cross-reactivity to Influenza A and B virus, RSV, Adenovirus, HBsAg, Syphilis, *H. pylori*, HIV, and HCV. The procedures followed the manufacturer’s instructions. A positive result was established when a colored line(s) appeared in the IgG or IgM regions, while a negative outcome consisted of no colored lines in these regions. In case no colored line appeared in the control region or when the intensity of the color in the IgG or IgM regions was low, the assays were repeated to ensure accuracy and reliability.

### Statistical analysis

LFIA results were collected on a database. Categorical variables were expressed in frequency and percentages. Statistical analyses were performed with the Statistical Package of Social Science (SPSS), version 25.0. Binary logistic regressions were used to calculate odds ratios (OR) and 95% confidence intervals (CI) for seropositivity comparisons between the different groups. *P-values* of < 0.05 were considered statistically significant.

### Chemiluminiscence immunoassay

The detection of anti-SARS-CoV-2 IgG/IgM antibodies via CLIA was carried out using a Maglumi 800 analyzer. This process followed the manufacturer’s protocol provided by SNIBE Ingelab for their assay.

### Electrochemiluminiscence immunoassay

The identification of antibodies targeting the SARS-CoV-2 Spike protein was performed on a Cobas Elecsys e411 analyzer. The assay followed the manufacturer’s protocol provided by Roche.

## Results

In the present study, the seroprevalence of anti-SARS-CoV-2 IgG/IgM antibodies was determined using a LFIA called Rapid 2019-nCoV IgG/IgM Combo Test Card (Colloidal Gold). Furthermore, to investigate the presence of anti-SARS-CoV-2 antibodies in the Ecuadorian population before the beginning of the restrictive measures, 1,018 samples were tested. As a result, five samples were positive in the LFIA; however, CLIA and ECLIA testing was subsequently performed, and the results were negative.

### Overall seroprevalence of anti-SARS-CoV-2 IgG/IgM antibodies

**Figure 1:**
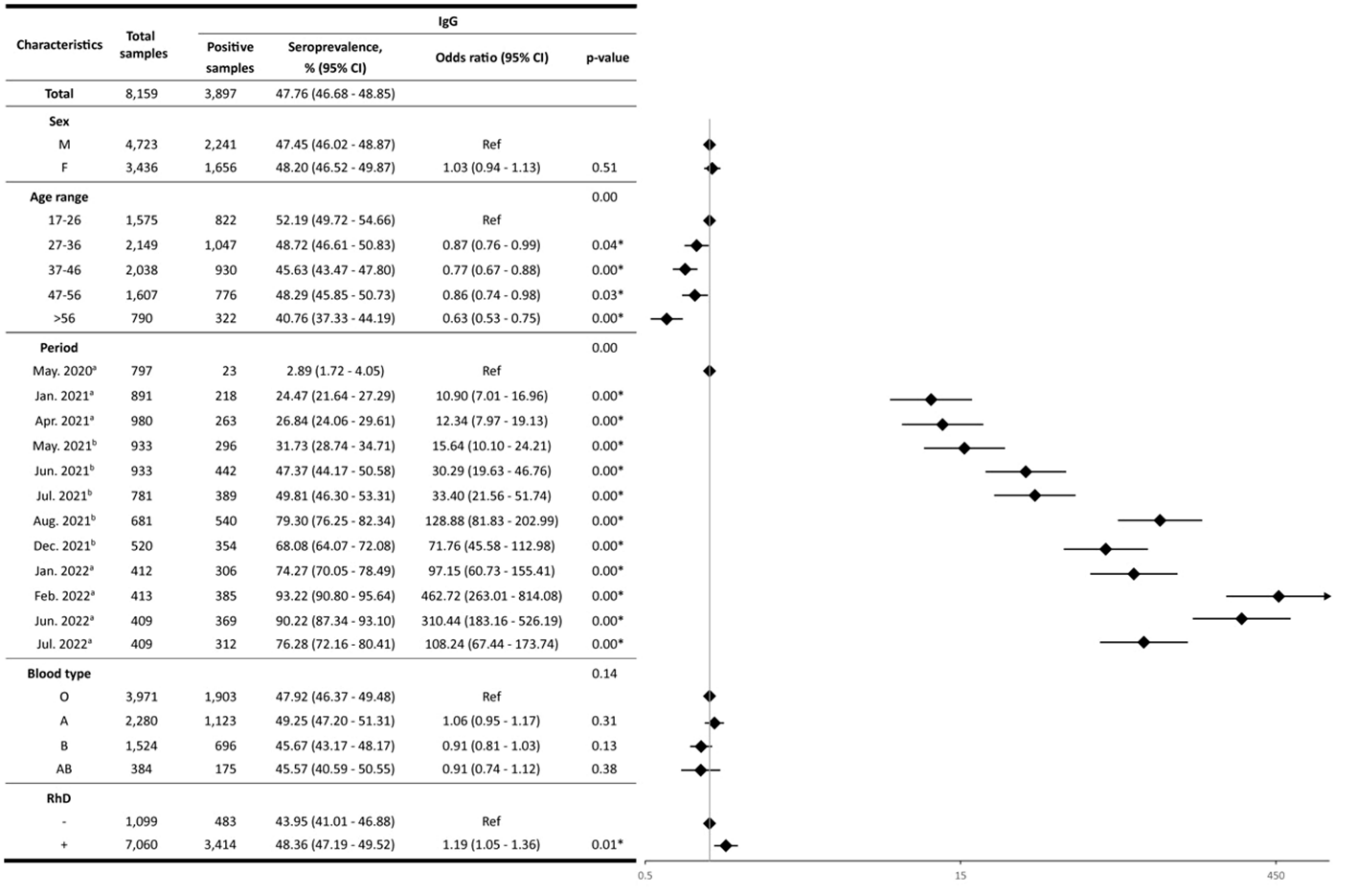
IgG seroprevalence. The forest plot (log scale) illustrates the Odds Ratio (OR) for IgG antibody seroprevalences across various characteristics. On the y-axis, characteristics like sex, age range, period, blood type, and RhD are represented, while the x-axis depicts the corresponding OR values. The rhombus box (black) represents the OR of each characteristic. The length of the horizontal line running across each rhombus represents the 95% CI for the OR for each characteristic. The neutral point, marked as “1” on the x-axis, serves as a reference point. A vertical line passing through this neutral point helps identify the characteristics under evaluation.; ^a^: months with the highest confirmed case rates; ^b^: months with the highest vaccination rates; *: statistically significant values.

**Figure 2:**
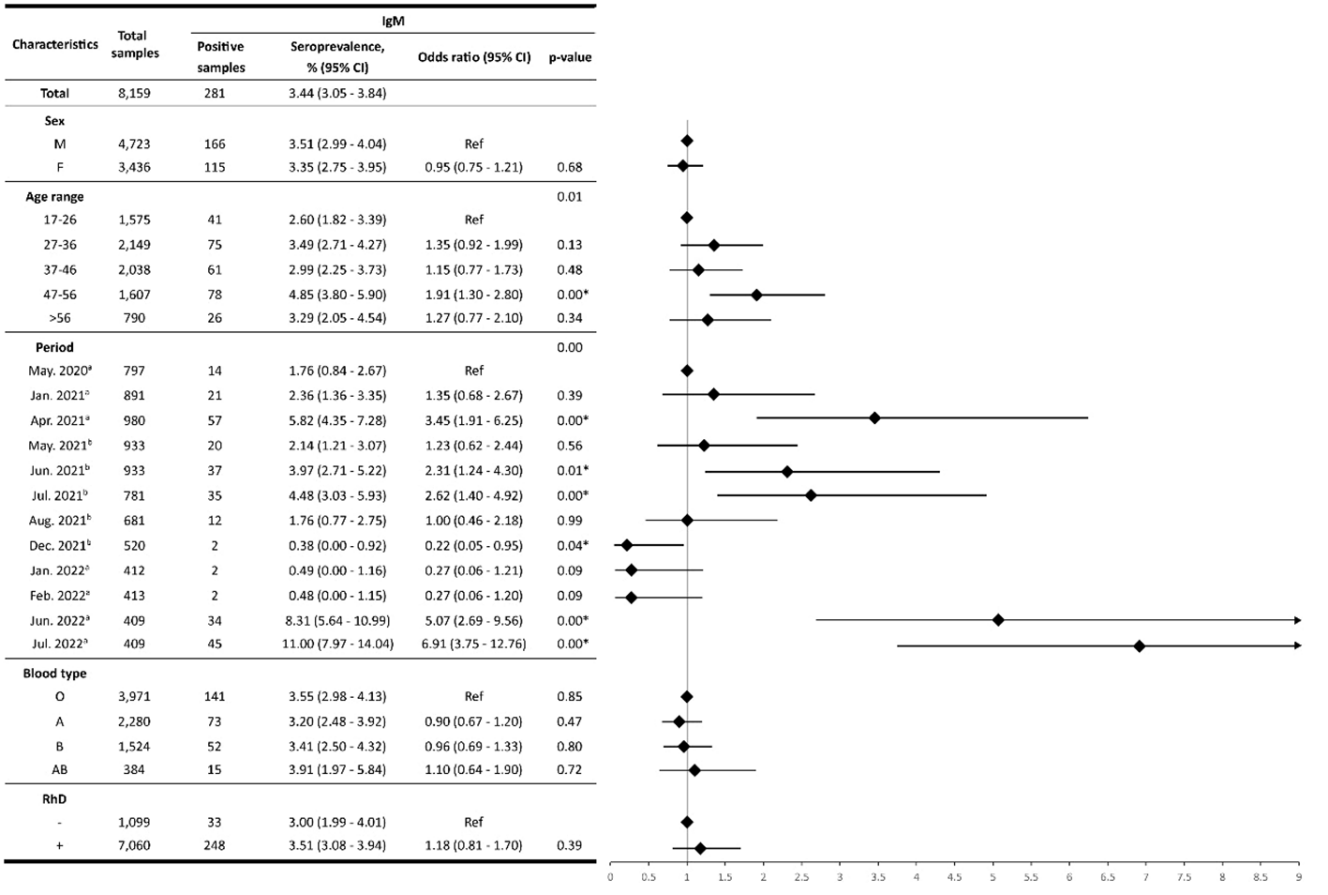
IgM seroprevalence. The forest plot (log scale) illustrates the Odds Ratio (OR) for IgM antibody seroprevalences across various characteristics. On the y-axis, characteristics like sex, age range, period, blood type, and RhD are represented, while the x-axis depicts the corresponding OR values. The rhombus box (black) represents the OR of each characteristic. The length of the horizontal line running across each rhombus represents the 95% CI for the OR for each characteristic. The neutral point, marked as “1” on the x-axis, serves as a reference point. A vertical line passing through this neutral point helps identify the characteristics under evaluation.; ^a^: months with the highest confirmed case rates; ^b^: months with the highest vaccination rates; *: statistically significant values.

Among the 8,159 samples, 3,897 tested positive for anti-SARS-CoV-2 IgG, and 281 for anti-SARS-CoV-2 IgM. Therefore, the overall IgG seroprevalence was 47.76%, whereas the overall IgM seroprevalence was 3.44% (**Figures 1**, **2**).

### Seroprevalence by sex

The study included 4,723 (57.89%) males and 3,436 (42.11%) females. Out of these, 2,241 and 1,656 yielded positive results for anti-SARS-CoV-2 IgG antibodies, respectively. Thus, the IgG seroprevalence for males was 47.45%, whereas for females, it was 48.20%. Moreover, the IgM seroprevalence in females was 3.35%, and for males, 3.51%. No statistically significant differences (*p-value*>0.05) were found between both groups (**Figures 1**, **2**).

### Seroprevalence by age

The seroprevalence analysis based on age groups revealed notable patterns. The first group (17 to 26 years old) had the highest IgG seroprevalence with 52.19%, whereas the fourth group (47 to 56 years old) showed the highest IgM seroprevalence with 4.85%. IgM seroprevalence results for the different age groups were not statistically significant, except for the fourth group, which showed a 1.91-fold higher probability of seropositivity in comparison with the reference group aged 17-26 years (95%CI: 1.30 - 2.80, *p-value*<0.05) (**Figure 2**). In contrast, significant differences were observed for IgG seropositivity in the age groups of 27-36 years (OR:0.87, 95%CI: 0.76-0.99), 37-46 years (OR:0.77, 95%CI: 0.67-0.88), 47-56 years (OR:0.86, 95%CI: 0.74-0.98) and > 56 years (OR:0.63, 95%CI: 0.53-0.75), compared to reference group (p-value<0.05) (**Figure 1**).

### Seroprevalence by periods

**Figure 3:**
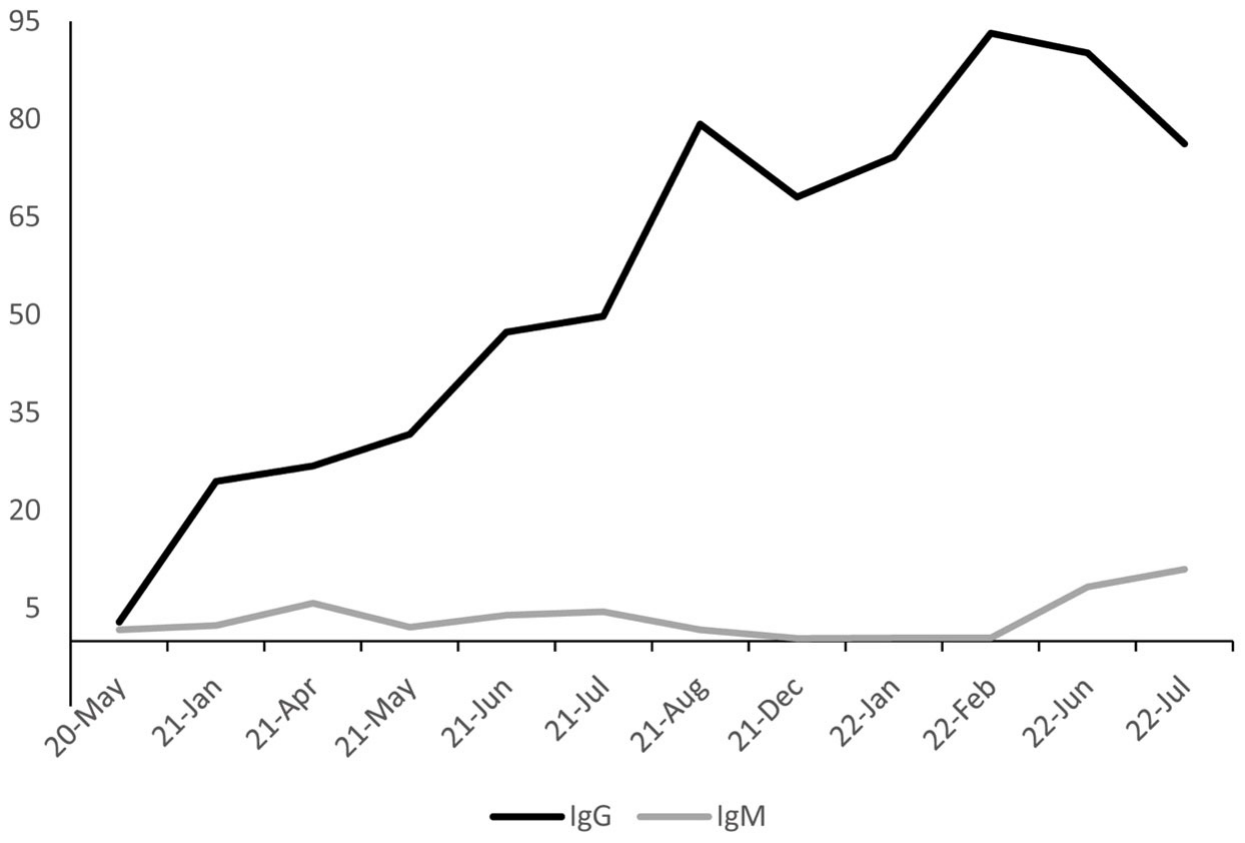
Anti-SARS-CoV-2 IgG and IgM seroprevalence trends. The IgG seroprevalence on the analyzed periods is presented with a black line, while the IgM seroprevalence is depicted with a grey line; ^a^: months with the highest confirmed case rates; ^b^: months with the highest vaccination rates.

A comparative analysis of seroprevalence was performed for each period (**Figure 3**). The IgM seroprevalence was the highest in the July 2022 period, reaching 11%. Furthermore, the results showed significant differences in IgM seroprevalence (*p-value*< 0.05) in April 2021 (OR:3.45, 95%CI: 1.91-6.25), June 2021 (OR:2.31, 95%CI: 1.24-4.30), July 2021 (OR:2.62, 95%CI: 1.40-4.92), December 2021 (OR:0.22, 95%CI: 0.05-0.95), June 2022 (OR:5.07, 95%CI: 2.69-9.56), and July 2022 (OR:6.91, 95%CI: 3.75-12.76) periods. In these periods, the odds of harboring IgM were higher than the reference group with the exception of December 2021. Conversely, in January 2021, May 2021, August 2021, January 2022, and February 2022, no significant differences compared to the reference group were found (*p-value*>0.05) (**Figure 2**).

Moreover, the results showed a gradual increase in IgG seropositivity over time (*p-value*<0.05). Between May 2020 and August 2021, IgG seroprevalence increased from 2.89% to 79.30% (OR: 128.88, 95% CI: 81.83 - 202.99). However, IgG seroprevalence dropped to 68.08% in December 2021 (OR: 71.76, 95%CI: 45.58-112.98). Subsequently, IgG seroprevalence rose again from January 2022 (74.27%) to February 2022 (90.22%) (OR: 97.15, 95%CI: 60.73-155.41; OR: 462.72, 95%CI: 263.01-814.08), followed by a slight decline in June 2022 (90.22%) and a more pronounced decrease in July 2022 (76.28%) (OR: 310.44, 95%CI: 183.16-526.19; OR: 108.24, 95%CI: 67.44-173.74).

### Seroprevalence by blood type

The IgG seroprevalence within the O, A, B, and AB blood groups was 47.92% (95%CI: 46.37 - 49.48), 49.25% (95% CI: 47.20 - 51.31), 45.67% (95% CI: 43.17 - 48.17), and 45.57% (95% CI: 40.59 - 50.55), respectively. Blood type A exhibited the highest IgG seroprevalence. Nevertheless, no significant differences (*p-value*>0.05) were found among blood groups (**Figure 1**).

Moreover, the IgM seroprevalence within the O, A, B, and AB was 3.55% (95% CI: 2.98 - 4.13), 3.20% (95% CI: 2.48 - 3.92), 3.41% (95% CI: 2.50 - 4.32), and 3.91% (95% CI: 1.97 - 5.84), respectively. Blood group AB had the highest prevalence of IgM antibodies. However, the IgM seroprevalence did not show significant differences (*p-value*>0.05) (**Figure 2**).

Regarding RhD, the IgG seroprevalence within the Rh+ group was 48.36% (95% CI: 47.19 - 49.52), while for the Rh- group was 43.95% (95%CI: 41.01-46.88). Noteworthily, it was determined that Rh+ individuals had an increased likelihood of carrying anti-SARS-CoV-2 IgG antibodies (OR:1.19, *p-value*<0.05) (**Figure 1**). On the other hand, no significant differences were found for the anti-SARS-CoV-2 IgM antibodies (*p-value >*0.05) (**Figure 2**).

## Discussion

Following the declaration of the pandemic, the World Health Organization (WHO) and several developed countries launched a massive vaccination plan to reduce mortality and infection rates [World Health Organization (WHO), 2022]. After the implementation of this vaccination program, infection and death rates decreased globally. Up until December 2023, more than 13,5 billion SARS-CoV-2 vaccine doses have been administered worldwide, with approximately 39 million doses in Ecuador (Mathieu et al., 2020b). In Ecuador, the most frequently administered vaccines in the general population were Sinovac Biotech (viral inactivated vaccine) (49.05% doses), Pfizer/BioNTech (mRNA vaccine) (28.11% doses), Oxford/AstraZeneca (viral vector vaccine) (20.91% doses), and CanSino Biologics (viral vector vaccine) (1.93% doses) (Ministerio de Salud Pública, 2022). Furthermore, data from Our World in Data indicates that more than 85.2% of the Ecuadorian population has been fully vaccinated (Mathieu et al., 2020b). Research has shown that herd immunity requires 70-85% of the population to be vaccinated (Suryawanshi and Biswas, 2023). Consequently, these statistics may suggest that herd immunity may have been reached in Ecuador because of vaccination campaigns.

In Ecuador, information regarding the seroprevalence of IgG and IgM anti-SARS-CoV-2 antibodies is scarce. Only a few studies have analyzed seroprevalence, and they were limited to specific regions. For instance, Acurio F. et al. (2021) conducted a cross-sectional study (n: 2,457) to determine the IgG and IgM seroprevalence in Cuenca, Ecuador. The authors reported a seropositivity of 11% (Acurio-Páez et al., 2021). Similarly, Vallejo-Janeta A. et al. (2022) carried out a retrospective analysis (n:1,259) of the IgG seroprevalence in Esmeraldas, Ecuador. They observed an overall IgG seroprevalence of 11.68% in the region (Vallejo-Janeta et al., 2022). Likewise, Villacrés-Granda B. et al. (2022) analyzed the presence of IgG anti‐SARS‐CoV‐2 S1 subunit antigens of the viral spike protein in 88 convalescent plasma samples from 1.2 to 10.0 months. The authors found a seropositivity of 97.7% (86 of 88) (Villacrés-Granda et al., 2022).

The overall IgG seroprevalence in our study was 47.76% (95%CI: 46.68-48.85), indicating acquired immunity resulting from vaccination or prior SARS-CoV-2 infection. Moreover, the high IgG seroprevalence in February and June 2022 suggests that herd immunity may have been achieved, as more than 67% of the community presents anti-SARS-CoV-2 antibodies (Randolph and Barreiro, 2020). Furthermore, the high prevalence of IgG antibodies in February 2022 could be attributed to the introduction of the Omicron variant into Ecuador in December 2021 and the high rate of confirmed cases in January 2022 (Carrazco-Montalvo et al., 2022a; Carrazco-Montalvo et al., 2022b).

The decrease in IgG seropositivity observed in December 2021 and July 2022 could be attributed to a decline in antibodies against SARS-CoV-2, which may not be detected by LFIA. Brisotto et al. (2021) evaluated the IgG seroprevalence in healthcare workers at 1 and 4 months after complete vaccination with either BNT162b2 or mRNA-1273. The investigators found that after four months, the antibody titer decreased significantly from 559.8 AU/mL to 92.7 AU/mL (p < 0.001) (Brisotto et al., 2021).

Moreover, Sauré et al. (2023) assessed IgG seropositivity in 101,070 Chilean individuals. Among them, 16,014 had not received any vaccine, while 65,902 had received a primary vaccination series with CoronaVac, 18,548 with BNT162b2, and 606 with ChAdOx1. The researchers established a decline in IgG seropositivity among individuals who received CoronaVac at week 18 and those who received BNT162b2 at week 25 (Sauré et al., 2023). These findings agree with those described in the present study, suggesting that the decrease in IgG levels may associated with the reduction of the induced immunity conferred by the first and second dose (August 2021) and the booster dose (January and February 2022).

In the present study, the IgM seroprevalence increased in April, June, and July 2021. This trend aligns with the high COVID-19 positivity rate reported by the MSP (39.6%, 26.5%, and 26.1%) during these periods (Ministerio de Salud Pública, 2020; Ministerio de Salud Pública del Ecuador, 2020), which are eight and five times higher than the WHO recommendations (Observatorio social del Ecuador, 2021). Furthermore, Delta variant cases were reported in Ecuador in the July 2021 period (PAHO/WHO, 2021), and this variant has been associated with a high contagious rate (Chavda et al., 2022), which could explain the increased IgM seroprevalence observed in this period. In early January 2022, the Omicron variant became the predominant strain in Ecuador, corresponding with a notable surge in case rates during that month (2.52%) (Guevara et al., 2022). Interestingly, despite that the MSP reported high infection rates in January and February 2022 due to the emergence of the Omicron variant, no association was found between IgM seropositivity and SARS-CoV-2 active infections in these periods (Mathieu et al., 2020b; Ministerio de Salud Pública, 2020).

According to the reports of COVID-19 cases by RT-qPCR, Ecuador experienced a peak positivity rate of 30.6% in June 2022, which continued until July 2022 (Ministerio de Salud Pública del Ecuador, 2020). This trend aligns with the results of the present study, which observed an increased IgM seroprevalence during these periods. The rise in COVID-19 cases could be attributed to a decreased COVID-19 vaccination rate and the reduction of protective measures (Observatorio social del Ecuador, 2021). Therefore, the results suggest a correlation between IgM seroprevalence in blood donor samples and the high rate of COVID-19 cases reported in Ecuador during the analyzed periods. However, this IgM trend must be interpretated carefully due to it could be attributed to previous vaccination or variable performance of the LFIA assays used in this study (Ortiz-Prado et al., 2020).

Another important variable to consider is the ABO blood groups, which have been associated with the susceptibility and severity of various disorders (Batool et al., 2017). Additionally, Zhao et al. (2020) were among the first to report an association between blood type and SARS-CoV-2 infection. The authors analyzed 2,173 samples from COVID-19 patients in China and found that blood type O was significantly correlated with a lower COVID-19 infection risk, whereas blood type A was related to a higher COVID-19 infection predisposition (Zhao et al., 2021). Similarly, other studies have reported consistent findings regarding this correlation (Garibaldi et al., 2022). However, it is important to note that contradictory research conducted by Gamboa-Aguilar J. et al. (2022) described a significant correlation between blood type O and COVID-19 in the Mexican population (n: 5,000 blood donors) (Gamboa-Aguilar et al., 2022).

The susceptibility to SARS-CoV-2 infection in the A blood group has been linked to the absence of anti-A antibodies. This absence could facilitate the interaction between the SARS-CoV-2 S protein and the host cell receptor ACE2 (angiotensin-converting enzyme 2) (Jackson et al., 2022).

In the present study, no statistically significant differences were found between ABO blood groups and the presence of anti-SARS-CoV-2 IgG and IgM antibodies (**Figures 1**, **2**). This lack of difference could be attributed to the high vaccination rate observed after July 2021 (Ministerio de Salud Pública, 2022). Consequently, vaccine administration led to increased antibody production, resulting in similar seroprevalence rates among individuals with each blood group. This, in turn, may have contributed to limiting the spread of the disease.

The RhD antigen has also been related to SARS-CoV-2 susceptibility. For instance, Anderson JL. et al. (2022) conducted a study (n:180,564) that analyzed the correlation between the RhD antigen and COVID-19 risk. The authors reported a positive risk association between SARS-CoV-2 predisposition and the presence of the RhD antigen (Anderson et al., 2022).

In alignment with these findings, the results of the present study found a significant association between the presence of the RhD antigen and anti-SARS-CoV-2 IgG antibodies. Even though the molecular mechanisms have not been described yet, it can be hypothesized that the presence of RhD antigen may increase viral entry similarly to the blood-type antigens. These results are particularly important in the context of Ecuador, given that most of the population carries the RhD antigen (Núñez Cifuentes, 2022), which could lead to an increased SARS-CoV-2 predisposition. Conversely, no association was observed regarding IgM seroprevalence.

Furthermore, in the period prior to the beginning of the restrictive measures (January-March 2020, n=1,018), two plasma samples tested positive for IgG SARS-CoV-2 antibodies, and three for IgM. These specimens were subsequently re-analyzed using CLIA and ECLIA assays, resulting in negative outcomes. These findings agree with what has been documented previously in LFIA tests (Kharlamova et al., 2021). This highlights the importance of conducting a thorough assessment of the immunological assays available in Ecuador to ensure their quality and accuracy.

### Public health implications

The public health implications include understanding the extent of COVID-19 in the Ecuadorian population and its associations with risk factors like blood type. Moreover, the study suggests that herd immunity may have been achieved based on the proportion of antibodies, emphasizing the importance of monitoring, and adapting public health strategies accordingly.

Despite the lack of statistical significance in the study’s outcomes, the potential association between blood types, the RhD antigen, and COVID-19 predisposition presented here should be further investigated. This information can be useful to identify high-risk populations and adjust public health interventions.

The implications of these findings extend beyond Ecuador, as they could be extrapolated to other regions with similar characteristics, assisting in the formulation of effective public health strategies and vaccine distribution plans.

### Limitations

This study acknowledges various limitations that could impact the generalizability of its results. Primarily, the cohort is comprised of healthy individuals. Consequently, the seroprevalence observed in this group may not accurately reflect the seroprevalence in populations with underlying health conditions such as cancer, chronic diseases, or those hospitalized. Furthermore, the study did not include children or the elderly. These groups may exhibit different immune responses and, consequently, different levels of seropositivity for anti-SARS-CoV-2 IgG and IgM antibodies.

The geographical representation of the data is another limitation. The seropositivity rates reported may not be indicative of all regions within Ecuador, particularly rural areas. A significant majority of the samples, 64.86% (n=5952), originated from the province of Pichincha. Other contributions included 10.32% (n=947) from Manabí, 10.30% (n=945) from Santo Domingo de la Tsáchilas, 6.02% (n=552) from Imbabura, 3.61% (n=331) from Carchi, and 2.22% (n=204) from El Oro. The remaining samples were collected from Napo (n=84), Zamora Chinchipe (n=73), Orellana (n=51), and Esmeraldas (n=37). Moreover, there are no statistically significant differences in seropositivity rates among the highlands, coastal, and Amazonian regions (Data not shown).

Another limitation is that the anonymization of samples resulted in restricted access to clinical data. Thus, the study could not correlate seropositivity with detailed clinical histories. Lastly, due to the constrained situation in the country, the epidemiological context was not fully captured.

## Conclusion

The present study aims to fill a gap in the existing literature by providing data on the seroprevalence of IgG and IgM anti-SARS-CoV-2 antibodies in Ecuador. Additionally, it sheds light on the positive impact that vaccination has had, showing that by February and June 2022, more than two-thirds of the community displayed evidence of acquired immunity, indicating that herd immunity has been reached.

Likewise, the project shows the dynamics of seroprevalence over several periods and suggests a possible influence between blood types, the RhD antigen, and COVID-19 susceptibility. These findings have implications for public health strategies and vaccine distribution in Ecuador and potentially in other regions with similar characteristics.

## Data availability statement

The original contributions presented in the study are included in the article/**Supplementary Material**. Further inquiries can be directed to the corresponding author.

## Ethics statement

The studies involving humans were approved by Human Beings Research Ethics Committee of Universidad UTE (code number: CEISH-2022-033). The studies were conducted in accordance with the local legislation and institutional requirements. The ethics committee/institutional review board waived the requirement of written informed consent for participation from the participants or the participants’ legal guardians/next of kin because each sample’s data was anonymized to ensure that no identifiable information was included. This anonymization process aimed to protect the privacy and confidentiality of the participants.

## Author contributions

AG: Conceptualization, Data curation, Resources, Writing – review & editing. RT-T: Conceptualization, Data curation, Methodology, Writing – original draft. EP-C: Conceptualization, Data curation, Methodology, Writing – original draft. SC-U: Conceptualization, Data curation, Investigation, Writing – original draft. PG-R: Data curation, Methodology, Visualization, Writing – original draft. VR-P: Data curation, Methodology, Writing – original draft. FC: Data curation, Resources, Writing – review & editing. VA-T: Data curation, Resources, Writing – review & editing. KR: Data curation, Validation, Writing – review & editing. MY: Data curation, Validation, Writing – review & editing. AC-A: Data curation, Writing – review & editing. AKZ: Conceptualization, Data curation, Project administration, Resources, Writing – original draft, Writing – review & editing.
