# Supplementary material for: Assessment of the COVID-19 pandemic progression in Ecuador through seroprevalence analysis of anti-SARS-CoV-2 IgG/IgM antibodies in blood donors
Source: Front Cell Infect Microbiol. 2024 Jun 21;14:1373450. doi: 10.3389/fcimb.2024.1373450 (PMC11224293; doi:10.3389/fcimb.2024.1373450)
Supplement: Supplementary file 1 [file Table_1.docx]

Supplementary Table 1. Information regarding points of collection, national epidemiology status, and sample size.

| **Point of collection** | **National epidemiology status** | **Sample Size** |
| --- | --- | --- |
| May 2020 | First infection wave | 797 |
| Jan 2021 | Second infection wave | 891 |
| April 2021 | Third infection wave | 980 |
| May 2021 | Before vaccination campaign | 933 |
| June 2021 | Before vaccination campaign | 933 |
| July 2021 | First vaccine dose | 781 |
| Aug 2021 | Second vaccine dose | 681 |
| Dec 2021 | Booster vaccine dose | 520 |
| Jan 2022 | Booster vaccine dose / Fourth infection wave | 412 |
| Feb 2022 | Fourth infection wave | 413 |
| June 2022 | Fifth infection wave | 409 |
| July 2022 | Fifth infection wave | 409 |
